# Integrated MRSA-Management (IMM) with prolonged decolonization treatment after hospital discharge is effective: a single centre, non-randomised open-label trial

**DOI:** 10.1186/s13756-016-0124-5

**Published:** 2016-06-14

**Authors:** Bernhard Jahn, Trudy M. Wassenaar, Annemarie Stroh

**Affiliations:** Frankfurter Diakonie Kliniken, Wilhelm-Epstein-Strasse 4, Frankfurt am Main, D-60431 Germany; AGAPLESION HYGIENE, Institute of Hygiene and Environmental Medicine, Ginnheimer Landstrasse 86, Frankfurt am Main, D-60487 Germany; Molecular Microbiology and Genomics Consultants, Tannenstrasse 7, Zotzenheim, D-55576 Germany

**Keywords:** MRSA, Methicillin resistance, Decolonization, Polyhexanide, Hospital, Domestic, Long-term care, Length of stay, LOS

## Abstract

**Background:**

Guidelines for the control of hospital-acquired MRSA include decolonization measures to end MRSA carrier status in colonized and infected patients. Successful decolonization typically requires up to 22 days of treatment, which is longer than the average hospital length of stay (LOS). Incomplete decolonization is therefore common, with long-term MRSA carriage as a consequence. To overcome this, we developed an integrated MRSA Management (IMM) by extending MRSA decolonization to the outpatient and domestic setting. The protocol makes use of polyhexanide-based products, in view of reported *qac*-mediated resistance to chlorhexidine in *S. aureus* and MRSA.

**Methods:**

This is a prospective, single centre, controlled, non-randomized, open-label study to evaluate the efficiency of the IMM concept. The outcome of guideline-approved decolonization during hospital stay only (control group; *n* = 201) was compared to the outcome following IMM treatment whereby decolonization was continued after discharge in the domestic setting or in a long-term care facility (study group; *n* = 99). As a secondary outcome, the effect of MRSA-status of skin alterations was assessed.

**Results:**

The overall decolonization rate was 47 % in the IMM patient group compared to 12 % in the control group (*p* < 0.01). The continued treatment after hospital discharge was as effective as treatment completed during hospitalization, with microbiologically-confirmed decolonization (patients with completed regimes only) obtained with 55 % for the IMM group and 43 % for the control group (*p* > 0.05). For patients with skin alterations *(e.g.* wounds and entry sites), decolonization success was 50 % if the skin alterations were MRSA-negative at baseline, compared to 22 % success for patients entering the study with MRSA-positive skin alterations (*p* < 0.01).

**Conclusions:**

The IMM strategy offers an MRSA decolonization protocol that is feasible in the domestic setting and is equally effective compared with inpatient decolonization treatment when hospital LOS is long enough to complete the treatment. Moreover, for patients with average LOS, decolonization rates obtained with IMM are significantly higher than for in-hospital treatment. IMM is a promising concept to improve decolonization rates of MRSA-carriers for patients who leave the hospital before decolonization is completed.

**Electronic supplementary material:**

The online version of this article (doi:10.1186/s13756-016-0124-5) contains supplementary material, which is available to authorized users.

## Background

Control and prevention of MRSA infections in a health-care setting is an ongoing challenge that requires critical infection control measures [1–3]. Various protocols have emerged over the years to prevent nosocomial transmission, and national guidelines for the control of MRSA in hospitals are now in place in many parts of the world (e.g. [4–6], reviewed in [7]). As part of these guidelines, decolonization of MRSA carriers is often recommended, since this can effectively reduce the risk of infection, in particular when performed prior to high-risk interventions such as surgery or central catheter insertion [8]. Universal decolonization of all patients has been shown to reduce bloodstream infections in an intensive care setting even more effectively in comparison to targeted decolonization of carriers only [9].

MRSA colonization is frequently persistent in adults, as demonstrated by the fact that approximately half of the patients tested positive upon admission were still positive one year later [10]. The decolonization procedure normally takes approximately 7 days, but when unsuccessful it needs to be repeated, and combined with the required culture diagnostics, this adds up to 15 to 20 days for a complete decolonization procedure. However, the average length of hospital duration is typically shorter than this and continues to decline over time; in 2009 the reported average LOS for acute care varied from in 18.5 days in Japan to 3.9 days in Mexico, with an average of 7.2 days for all OECD countries [11]. In Germany, the average LOS of 9.7 days in 2009 was decreased to 7.4 days in 2014 [12]. Clearly, short hospital stays hamper completion of decolonization cycles. Typically, the microbiological follow-up of inpatients is omitted so that the carrier status of patients at discharge is uncertain.

Unfortunately, a significant proportion of MRSA-positive admissions are readmissions; published data vary from 35 % [13] to 68 % [14]. In our experience approximately half of all MRSA-positive inpatients are readmissions. These patients have often been subjected to incomplete decolonization treatment during a previous hospital stay, in which case the procedure needs to be restarted upon readmission. For these patients, the risks related to MRSA carriage persist despite having experienced the discomfort of decolonization attempts, while for the hospital such ineffective decolonization efforts add to the MRSA-associated economic burden [15, 16]. Moreover, incomplete decolonization attempts can potentially select for resistant bacterial populations [17].

To overcome the limitations of MRSA decolonization of inpatients with short hospitalization duration, we developed a new strategy for an Integrated MRSA Management (IMM). An essential component of IMM is that MRSA decolonization and concomitant diagnostics does not terminate with discharge, but are continued in the patient’s domestic setting until successful decolonization has been demonstrated by microbiological culture.

The aim of the current prospective, single centre, controlled, non-randomized, open-label study was twofold: it was performed to assess if the IMM strategy was able to improve the overall success rate of MRSA decolonization, and, in addition, to compare the obtained results of IMM decolonization with success rates of inpatient decolonization only. The effect of MRSA-status of skin alterations was assessed as a secondary outcome. In view of reported chlorhexidine resistance in *S. aureus* as well as in MRSA due to acquired *qac* resistance genes [18, 19], and based on past experience with inpatient decolonization, we mostly applied polyhexanide-based products for the decolonization procedures in the inpatient control group as well as the IMM study group.

## Methods

### Study concept

**Fig. 1:**
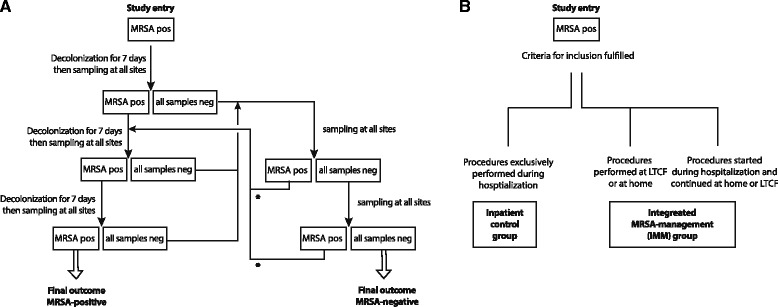
Schematic of the study. In panel (**a**), the procedure is shown, with arrows representing actions and boxes representing an MRSA status. The asterisks indicate that the decolonization treatment could be repeated with a maximum of 3 treatments in total. Depending on the number of required decolonization treatments (1 to 3) the procedure was completed in 11 to 25 days. Panel (**b**) shows the inpatient control group and the IMM group. The treatment regime was the same for both, though the location of the patients differed

A schematic of the study concept is shown in Fig. 1. The study was performed in a tertiary care teaching hospital in the Rhine-Main metropolitan area, Germany. Patients admitted to surgical as well as internal medicine units were considered for inclusion, with the exception of admissions to paediatrics and intensive care. For the inpatient control group (represented to the right of Fig. 1b), patients were recruited from 810 adults admitted to the study hospital between August 2007 and May 2009, who were positive with MRSA as established by screening upon entry or during diagnostic procedures, or with a known prior status (for readmissions). Out of these, 201 patients with a hospital stay of at least 7 days were chosen, which was the main inclusion criterion for the inpatient control group. Further, these patients were at least 18 years of age and, although identified as MRSA carriers, showed no symptoms of an acute MRSA infection. This resulted in a Full Analysis Set (FAS) of 201 patients for the inpatient control group, who received all decolonization procedures routinely used in the hospital, as described in the Additional file 1. Briefly, the nasal cavity was decolonized with mupirocin 3 times daily for 5 days, after which Prontoderm was used twice daily. All other body parts were treated on a daily basis with the Polyhexanide-based products listed in Table 1 (all products by B. Braun Medical AG, Sempach, Switzerland). Patient’s utensils and environment were disinfected or cleaned according to the hospital’s routine regime. The status of colonization was assessed every 7 days and the decolonization regime was terminated when an MRSA-free status was achieved (see below). Decolonization procedures were also terminated in case of discharge, irrespective of their MRSA status.

**Table 1:** Polyhexanide-based antiseptics used for decolonization

	Decolonization site	Polyhexanide concentration	Other ingredients
ProntOral®	throat, mouth wash	0.15 %	Aroma, sodium cyclamate, surfactants, excipients
Prontoderm® Nasal gel	nose	0.1 %	Glycerine, hydroxyethylcellulose, excipients
Prontosan® Solution	wound irrigation	0.1 %	Betaine surfactant
Prontoderm® Solution/Foam	whole-body/hair washing, external auditory canal (Solution only)	0.11 %	Surfactants, excipients

The criterion for The IMM group was that decolonization treatment was completely or partly performed in the outpatient setting (Fig. 1b). Patients were recruited for the IMM cohort during the same period as above, from either admissions (*n* = 62), or actively contacted based on their MRSA status that was known from previous admissions. The latter were recruited either from long-term care facilities (LTCF) (*n* = 12) or from domestic settings (*n* = 25); these patients received the complete treatment at LTCF or at home, performed by a nurse under supervision of hospital staff. This resulted in a FAS of the IMM group of 99 patients. The treatment regime was identical between inpatient control group and IMM group, other than the location of the IMM patients during (part of) their treatment.

The total study thus comprised 300 investigated patients, 182 male and 118 female, whose pre-existing skin alterations (*e.g*. wounds or catheter entry sites) were recorded by the visiting study nurse, for which personnel was appointed; this personnel was supervised by the overseeing infection control nurse.

According to national guidelines, MRSA decolonization procedures were regarded as successful when 3 consecutive sets of negative MRSA samples were obtained from a patient. The PPS of the inpatient control group comprised of those patients who were successfully decolonized, as well as patients who had completed the full decolonization procedure covering 22 ± 3 days, but who at the end produced fewer than 3 subsequent sets of negative MRSA-control samples obtained. This was scored as unsuccessful decolonization. In the IMM intervention group, six patients prematurely discontinued the study and nine additional patients were excluded due to schedule interruptions, thus resulting in a PPS of the intervention group of 84 patients who completed the decolonization procedure and follow-up, with the outcome of successful or unsuccessful decolonization, as defined above.

All procedures were documented on site by handheld-based data acquisition.

### Determining MRSA status and decolonization procedures

MRSA colonization status was determined by a series of swab samples taken from the nares, oral cavity/throat, ears/hairline and abdomen/groin as standard localizations (representing one sample series) and from representative skin alterations if applicable (e.g. wounds and catheter entry sites). The sampling regime is summarized in the Additional file 1. Microbiological culture was performed according to standard procedures. Briefly, cotton swabs with transport Amies media (Sarstedt, Germany) were moistened with sterile normal saline (B. Braun, Germany) prior to sampling. Each swab was transferred to 10 ml TSB broth culture (bioMérieux, Germany) and microbial growth was visually assessed by turbidity after 24 h and 48 h incubation. Samples without detectable growth after 48 h were considered negative. For those samples showing bacterial growth, cultures were plated on selective chromogenic media (Oxoid Brilliant MRSA Agar, Oxoid, Germany) for 18 - 24 h.

For initial MRSA-diagnostic, colonies morphologically corresponding to presumptive MRSA on chromogenic media were further validated by biochemical characterisation (Vitek 2, bioMérieux, Germany), presence of PBP2a (Slidex MRSA-detection, bioMérieux) and antibiotic testing (Vitek 2, bioMérieux). When morphology on chromogenic substrate and the biochemical tests were in accordance, subsequent samples of the same patient were considered MRSA-positive, corresponding with growth on the selective, chromogenic media.

The decolonization treatment was terminated in case the patient’s status changed to MRSA negative. All recommended elements of inpatient MRSA decolonization according to the national (German) guidelines [6] were adapted for the IMM group to be performed in the outpatient setting by home care nursing. Samples for MRSA screening were taken at the day of admission and, when all requirements were met, the patient was enrolled at day 1, receiving decolonization treatment. When all samples from a sample series were negative, treatment was stopped and sampling was immediately repeated (Fig. 1). Individual patients received a maximum of three decolonization treatments, giving a complete duration of 25 days at most. The primary efficacy variable was the rate of successful MRSA-decolonization in the IMM-study population compared to the control group.

All antiseptics used for decolonization contained polyhexanide (polyaminopropyl biguanide), as the active component, with the exception of mupirocin used for nasal treatment during the first 5 days. Standard hygiene measures, including wound care procedures were applied to all patients enrolled upon demand. All decolonization and wound care procedures were performed in accordance to national recommendations, accompanied by a daily decontamination/disinfection of items involved in patient’s personal hygiene, surfaces in the patient’s proximity, a daily change of bed linen, as well as a change of body clothes following each body washing. A chart of the practical procedures is available as supplementary information.

### Statistical analysis

The primary efficacy variable was compared using a Chi-Square test with error probability α = 0.05. Continuous and categorical variables are described by: number of valid cases, mean, median, standard deviation, minimum, maximum, and number of valid cases, frequency and percentage, respectively. All statistical analyses were performed using SAS® Version 9.1.3.

## Results

### Study population

The age and gender distribution of the study population is shown in Table 2; demographics did not differ significantly between IMM and control group. The FAS of the intervention group for the IMM study cohort comprised 99 MRSA-positive patients. After exclusion of 15 patients, for reasons described in the Methods, 84 patients were valid for the PPS (Table 2). A quarter of these patients had been admitted from home. Of the 62 patients who were recruited during hospitalization, 55 received MRSA decolonization treatment prior to discharge, varying from 2 to 21 days of duration. Twelve patients who were enrolled lived in long-term care facilities. The decolonization treatment was continued after discharge as required, which took place in the patient’s home in 77 cases and in long-term care in 22 cases (Table 2).

**Table 2:** Characteristics of the study participants

A. Demographic characteristics^a^		Interventional IMM-population	Inpatient control group
		(*N* = 99)	(*N* = 201)
Age		years	years
	Mean	68	71
	Standard deviation	±15	±13
	Range	27-96	20-95
Age category		*N* (%)	*N* (%)
	<65 years	35 (35 %)	56 (28 %)
	>65 years	64 (65 %)	145 (72 %)
Gender		*N* (%)	*N* (%)
	Male	60 (61 %)	122 (61 %)
	Female	39 (39 %)	79 (39 %)
Skin alterations		*N* (%)	*N* (%)
	With skin alterations	60 (61 %)	136 (68 %)
	Without skin alterations	39 (39 %)	65 (32 %)
B. Study entry description			
Patients with entry in the study from		*N* (%)	*N* (%)
	Hospital	62 (63 %)	Hospital inpatient, by default
	Long-term care	12 (12 %)	
	Patient’s home	25 (25 %)	
Discharge location (IMM only)			
	Hospital	0	not applicable
	Long-term care	22 (22 %)	
	Patient’s home	77 (78 %)	
Patients not completing		6 (6 %)	0
Protocol violations		9 (9 %)	0
Per protocol Set (PPS)		*N* = 84	*N* = 54^b^

^a^t-test (age): *p* > 0.05. Chi-square test (gender, skin alterations): *p* > 0.05

^b^PPS inpatient control group: LOS of 22 ± 3 decolonization days or three negative MRSA-swabs

The FAS of the inpatient control group represented 201 patients (total control group). These patients received decolonization treatment only during their hospitalization stay. Of these patients, 54 were valid for the PPS (control group with completed decolonization cycle and follow-up in the hospital).

### Overall decolonization results

Considering the complete patient groups (FAS), at the end of the study 46 patients of the intervention IMM group (47 %) had become MRSA-free, while 53 (53 %) had remained MRSA-positive. Of these, one patient produced two negative swabs only, resulting in an indecisive status which we screened as positive. In the control group 24 inpatients had become MRSA-free (12 %) while 177 remained positive (88 %) of which 59 were indecisive. The difference in decolonization performance between the two groups was statistically significant (*p* < 0.001). Table 3A summarizes the data for the FAS populations. A significant difference was also observed when the comparison was restricted to patients with skin alterations: 32 % turned MRSA-negative in the intervention IMM group compared to 12 % in the control group, or to patients without skin alterations (69 % for the intervention IMM group compared to 12 % for control group, in both cases *p* < 0.001).

**Table 3:** Obtained MRSA-status at the end of the study, FAS and PPS results

A. Full Analysis Set (FAS) of 300 subjects							
Final MRSA-status n (%)	Interventional IMM-population			Inpatient control group			Chi-square test for final MRSA-status
	(*N* = 99)			(*N* = 201)			
	W/o skin alterations	With skin alterations	Total	W/o skin alterations	With skin alterations	Total	IMM-population vs. inpatient control group
	*N* = 39	*N* = 60	*N* = 99	*N* = 65	*N* = 136	*N* = 201	
MRSA-free (3 neg. swabs series)	27	19	46	8	16	24	For all patients: *p* < 0.001
	(69 %)	(32 %)	(47 %)	(12 %)	(12 %)	(12 %)	
Two negative swabs series	0	1	1	8	11	19	For patients without skin alterations: *p* < 0.001
		(2 %)	(1 %)	(12 %)	(8 %)	(9 %)	
One negative swab series	0	0	0	15	25	40	
				(23 %)	(18 %)	(20 %)	
Remainder MRSA-positive	12	40	52	34	84	118	For patients with skin alterations: *p* < 0.001
	(31 %)	(67 %)	(52 %)	(53 %)	(62 %)	(59 %)	
Total MRSA-positve	12	41	53	57	120	177	
	(31 %)	(69 %)	(53 %)	(88 %)	(88 %)	(88 %)	
Chi-square test for final MRSA-status	Patients with and without skin alterations: *p* < 0.001			Patients with and without skin alterations: *p* > 0.05			
B. Per Protocol Set (PPS) of 138 subjects with completed decolonization + follow-up							
Final MRSA-status n (%)	Interventional IMM-population			Inpatient control group			Chi-square test for final MRSA-status
	(*N* = 84)			(*N* = 54)			
	W/o skin alterations	With skin alterations	Total	W/o skin alterations	With skin alterations	Total	IMM-population vs. inpatient control group
	*N* = 36	*N* = 48	*N* = 84	*N* = 14	*N* = 40	*N* = 54	
MRSA-free (3 neg. swabs series)	27	19	46	7	16	23	For all patients: *p* > 0.5
	(75 %)	(40 %)	(55 %)	(50 %)	(40 %)	(43 %)	
Two negative swabs series	0	0	0	5	6	11	For patients without skin alterations: *p* > 0.5
				(36 %)	(15 %)	(20 %)	
One negative swab series	0	0	0	0	7	7	
					(18 %)	(13 %)	
Remainder MRSA-positive	9	29	38	2	11	13	For patients with skin alterations: *p* > 0.5
	(25 %)	(60 %)	(45 %)	(14 %)	(27 %)	(24 %)	
Total MRSA-positve	9	29	38	7	24	31	
	(25 %)	(60 %)	(45 %)	(50 %)	(60 %)	(57 %)	
Chi-square test for final MRSA-status	Patients with and without skin alterations: *p* < 0.01			Patients with and without skin alterations: *p* > 0.05			

Table 3B summarizes the results for the PPS, when the analysis was restricted to patient populations with completed decolonization protocols and a microbiological follow-up, either in the IMM study group or the inpatient control group. This analysis resulted in similar success rates for both groups (intervention group 46/84 or 55 %; inpatient control group: 23/54 or 43 %, *p* > 0.05), indicating that continuation of IMM treatment after discharge can be as effective as when performed within the hospital. A comparison within the IMM population between patients with and without skin alterations resulted in a significantly better decolonization rate for the latter (*p* < 0.01), whereby patients without skin alterations represented the minority in both populations (36/84 or 43 % in the IMM-population and 14/54 or 26 % in the control group).

#### Patient demography, hospitalization, and body-site specific data

**Fig. 2:**
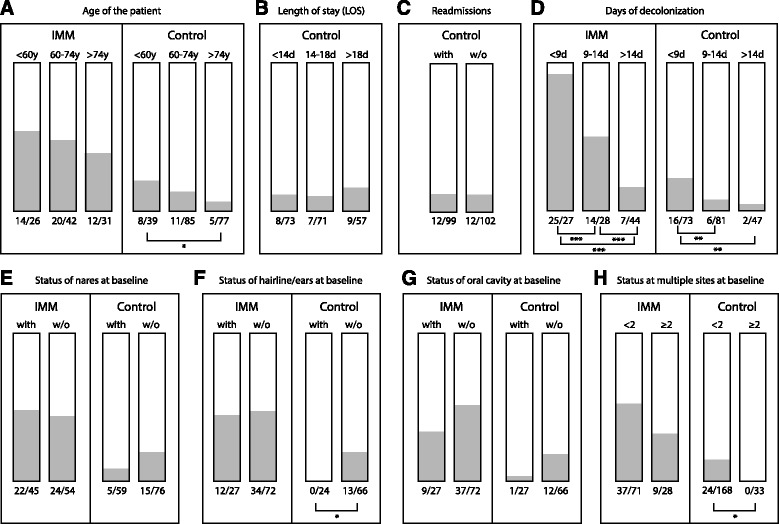
Analysis of patient demography, hospitalization and body-site specific data. The percentage of patients turned MRSA-negative is shown in grey, absolute numbers are shown below the column, *e.g.,* 14 turned negative out of 26 patients. Significance is indicated as *p < 0.05, **p < 0.01 and ***p < 0.001

Patient’s gender did not affect decolonization outcome, but as shown in Fig. 2 (panel a), with increasing age decolonization success decreased, both in the IMM and in the control group. The difference was significant for the control group only when comparing patients younger than 60 years to those over 74 years (*p* < 0.05).

The inhospital control group was used to assess the effect of LOS duration. This varied from 10 days to 28 days, with a mean of 16.1 days in the enrolled patients. Although longer LOS resulted in a higher percentage of decolonized patients, the result was not significant. Figure 2b shows the analysis for three cohorts with different LOS; a division for hospitalization shorter or longer than 14 days resulted in 12 % MRSA-free patients in both groups.

The inhospital control group comprised 99 patients who were readmissions. Of these, 12 turned MRSA-negative during the study (12 %), compared to 12 of 101 patients (12 %) who had not been hospitalized in the year previous to admission (*p* > 0.05) (Fig. 2c). Whether decolonization attempts had been performed during previous admissions was not recorded, but prior hospitalization did not affect the outcome of decolonization.

We further analysed whether the length of the decolonization procedure correlated with the outcome. The mean duration of decolonization was 13.6 days for the complete inpatient group and 10.7 days for the control group. Decolonization treatment was successful after 7.5 days on average for IMM patients and 7.4 days on average in the control group. Decolonization success decreased with its duration; Panel d of Fig. 2 shows the breakup into three groups. While 25 of 27 patients in the IMM turned negative after 9 days or less of decolonization, only 7 of 44 did so after treatment longer than 14 days (this difference was statistically significant, *p* < 0.001). For the inpatient control group, 16 of 73 patients turned negative within 9 days, but only 2 after longer than 14 days of treatment (*p* < 0.01). Prolonged decolonization (>14 days) resulted in 7 and 2 patients with an MRSA-free status in the IMM and control group, respectively.

Analysis of the data with respect of the site of colonization (irrespective of presence of skin alterations, which is discussed below) revealed that there was no significant difference between IMM and control group for site-specific positive results obtained at baseline. As expected, MRSA was most frequently detected in the nares (45 % in the IMM group compared to 44 % in controls), followed by the abdomen/groin (33 % and 32 %, respectively), hairline/ears (27 % for each group) and oral cavity/throat (27 % for the IMM and 29 % for the control group). The overall difference in decolonization performance between IMM and inpatient control group was also observed when site-specific colonization at baseline was analyzed. The only significant finding when analysing baseline data per body site was that control patients negative at hairline/ears at baseline were more frequently completely negative at the end of the study, which was found for 13 out of 66 patients (20 %, *p* < 0.05).

Patients who were proven positive at more than one body site at baseline were also separately analysed. For the IMM group, there was no difference in decolonization performance for these patients compared to cases with one site-specific MRSA-positive sample at baseline, while in the inpatient control group none of the 33 cases positive at 2 or more body sites had turned completely negative at the end of the study, compared to 24 cases turning to a negative status with fewer than 2 positive body sites at baseline (*p* < 0.05).

### Decolonization rates related to MRSA in skin alterations

**Fig. 3:**
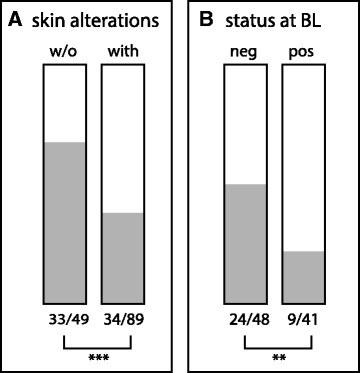
Effect of skin alterations on success rate of decolonization. Panel (**a**) shows the effect of absence or presence of skin alterations for PPS patients (intervention group and inpatient control group combined) upon admission. Panel (**b**) shows the effect of the MRSA-status of the skin alterations at baseline. (Significance **p < 0.01 and ***p < 0.001)

The secondary outcome of the study focused on the 138 patients who had finished the complete decolonization protocol irrespective of their location (in the hospital, *N* = 54 or after discharge, *N* = 84) to analyse the effect of skin alterations that had been present at baseline. Of 89 patients with skin alterations at the beginning of the procedure, 34 (38 %) had become MRSA-negative after completion of decolonization, while 33 of the 49 patients without skin alterations (67 %) had become negative (*p* = 0.001) (Fig. 3a). When present at the beginning of the study, the MRSA-status of such skin alterations was recorded. A success rate of 50 % was observed in patients with MRSA-negative skin alterations at the start of intervention (24 of 48), compared to a decolonization rate of 22 % in patients testing positive for MRSA in skin alterations (9 of 41) (*p* < 0.01) (Fig. 3b).

## Discussion

MRSA decolonization guidelines define a multimodal approach to abolish MRSA carriage. The impact of each component of the typical treatment is not fully understood. It has been reported that stay at ICU was the most strongly associated with decolonization success, while of the classical decolonization steps with nasal mupirocin treatment, chlorhexidine body wash and povidone-iodine wound treatment, the latter was most strongly correlated to success [20]. In another study, whole body wash with 4 % chlorhexidine solution for 5 days resulted in an eradication rate of 8 % only, while more extensive treatment was required for complete eradication of MRSA colonization [21]. An earlier study reported an 18 % eradication rate by chlorhexidine washing which was improved to 25 % when used in combination with mupirocin [22]. Various regimes using mupirocin for nasal decolonization, combined with octenidine, quaternary ammonium compounds or phenoxyethanol have also been reported to be effective against MRSA colonization [23–25].

Due to developed antibiotic resistance, alternatives to chlorhexidine are needed, for which data on efficacy are highly needed [26, 27]. Relatively few studies exist to date regarding MRSA-decolonization with polyhexanide, a product that is recommended for treating critically colonized, infected and chronic wounds [28]. It can be used in combination with antibiotic treatment or on its own and is equally effective against MRSA and methicillin-susceptible *S. aureus* [29]. Moreover, polyhexanide has been suggested as an alternative agent for decolonization of mupirocin-resistant (*mupA*-carrying) or chlorhexidine-resistant (*qac*-carrying) strains [30]. Disappointing results, namely 33.8 % decolonization, were obtained when polyhexanide was applied in a double-blind placebo-controlled trial in a teaching hospital [31]. In that study, treatment was performed for 7 days only, while successful decolonization was defined by negative samples at the end of treatment. We believe that a single polyhexanide-based decolonization course can be insufficient for MRSA-positive patients, and our results support this view. In addition, although the placebo-group in the study by Landelle and co-workers used the product without polyhexanide, another ingredient in the placebo formulation was shown to have an unexpected anti-staphylococcal activity [32], hence possibly reducing the difference observed between treatment and placebo group. That study also included decolonization treatment in outpatient settings, for which even fewer studies exist. Here, the impact of insufficient compliance on decolonization rate remained an open question [31].

In our study, the rate of over 50 % successful MRSA-decolonization seen in the PPS groups for both the inpatient as well as the IMM population indicates that a polyhexanide-based MRSA-decolonization treatment also provides a suitable approach to eradicate MRSA. In accordance to our results, the long-term care patients studied by Wendt and co-workers resulted in a similar outcome of decolonization success compared to the hospital inpatient group [21].

The presence of skin alterations has been recognized as a risk factor for decolonization failure [20, 24], though this notion was contradicted by others [25]. Our data confirm that the presence of wounds and skin entries can hamper decolonization, although the effect is relatively minor for skin alterations that are free of MRSA: a success rate of 75 % MRSA eradication in IMM patients without skin alteration was reduced to 50 % when skin alterations were negative for MRSA. The success rate was lower when the skin alterations were MRSA-positive at base line (18 %). These results underline the importance of wound care in MRSA management, as recently discussed by Meyer and co-workers [33]. A pilot study evaluating polyhexanide-mediated eradication of MRSA in chronic leg ulcers reported that eradication of MRSA in chronic wounds in outpatients is indeed possible [34].

There are several limitations to our study. Since this work was performed in and supervised from a single hospital, external validity of the results is not available. Although we believe that the IMM protocol can be easily adapted to other hospitals, generalization of the study results depends on practicalities and local habits that may affect the outcome. Moreover, patients were not followed-up to assess the long-term effects of decolonization. In addition, although readmissions were recorded, any previous decolonization attempts performed in other hospitals, the used products and their outcomes were not available for analysis. Further, data on body-site specific colonisation status at baseline were not always complete, so that analysis of the effects of these must be interpreted with care. Finally, transient colonization may have influenced our outcome. Transient colonization has been described for patients in LTCF as well as health care workers (e.g., [35]), but to our knowledge no data are available for a comparison between LTCF and in-house patients. Therfore, we do not know whether or to what degree transient colonization would affect the inpatient and control groups.

Our work builds on these data and shows that continuing the decolonization regime after hospital discharge can increase the success rate. The IMM strategy presented here offers an MRSA-decolonization protocol that is equally effective in an outpatient setting as is long-term inpatient decolonization treatment. In particular, the IMM strategy is recommended for short LOS patients. This study is the first to compare a combination of in- and outpatient MRSA-decolonization including treatment in a domestic setting, with a control group of patients undergoing decolonization whose duration is limited to the hospital stay.

## Conclusions

The work reported here provides proof of concept for a new and effective MRSA decolonization strategy, whereby decolonization can successfully be continued after hospital discharge. The study highlights the decolonization procedure as a pivotal treatment, which should be independent of the medical institution and solely dependent on the colonization status of the patient.

## Abbreviations

FAS, full analysis set; ICH-GCP, International Council for Harmonisation of Technical Requirements for Pharmaceuticals for Human Use-Good Clinical Practice; ICU, intensive-care unit; IMM, Integrated MRSA-Management; LOS, length of stay; LTCF, long-term care facility; MRSA, Methicillin-resistant *Staphylococcus aureus*; OECD, Organisation for Economic Co-operation and Development; PCR, polymerase chain reaction; PPS, per protocol set

## Additional file

Additional file 1: Decolonization protocol. (DOCX 18 kb)
