# Supplementary material for: Integrated MRSA-Management (IMM) with prolonged decolonization treatment after hospital discharge is effective: a single centre, non-randomised open-label trial
Source: Antimicrob Resist Infect Control. 2016 Jun 14;5:25. doi: 10.1186/s13756-016-0124-5 (PMC4908775; doi:10.1186/s13756-016-0124-5)
Supplement: Additional file 1: — Decolonization protocol. (DOCX 18 kb) [file 13756_2016_124_MOESM1_ESM.docx]

Supplementary Table: Decolonization protocol.

| **A. Patient care** | **Week 1** | | | | | **S2 (one negative sample series already obtained)** | **S3 (two negative sample series already obtained)** | **Day 23** |
| --- | --- | --- | --- | --- | --- | --- | --- | --- |
| **Body site** | **Action** | **Day 1** | **Days 2 to 5** | **Day 6** | **Day 7 onwards** |  |  | Terminate treatment |
| Nose, mouth, skin alterations, groin, ears and hairline, urine as required | Samples all body sites with moist swab**^1^** | Take samples on Day 1 |  |  | Take samples on Day 7 or 14 | Take samples after last negative result |  |  |
|  | **Check samples diagnostics** | ⇒  ⇒ | **S1.** When all negative, terminate treatment and  go to **S2** | | ⇒  ⇒ | When all negative**,** repeat sampling and go to **S3** | When 3 sets of negative samples are obtained**,** record patient as MRSA-negative |  |
|  |  |  | When ≥1 pos. continue treatment as for Day 6 | |  | When ≥1 sample positive go back to Day 6 regime **^2^** | When ≥1 sample positive go back to Day 6 regime **^2^** |  |
| Nose, nasal cavity | Apply muporicin | 3x daily | 3x daily |  | | | | |
|  | Apply Prontoderm Gel light |  | | 2x daily | 2x daily |  | | |
| Mouth | Mouthwash with ProntOral | 3x daily | 3x daily | 2x daily | 2x daily |  |  |  |
| Ears/Ear canal | Apply Prontoderm | 1x daily | 1x daily | 1x daily | 1x daily |  |  |  |
| Hair | Wash with Prontoderm/foam | 1x daily | 1x daily | 1x daily | 1x daily |  |  |  |
| Body | Wash with Prontoderm/foam | 1x daily | 1x daily | 1x daily | 1x daily |  |  |  |
| Hands | Desinfect with Softaman | as required | as required | as required | as required |  |  |  |
| Tracheostoma entry site | Apply Prontosan | 1x daily | 1x daily | 1x daily | as required**^3^** |  |  |  |
| Wounds | Apply Prontosan | 1x daily | 1x daily | 1x daily | as required**^3^** |  |  |  |
| Scars, closed stiched wounds | Apply Softasept N | 1x daily | 1x daily | 1x daily | as required**^3^** |  |  |  |
| Utensils, surfaces related to wound care | Desinfect with Meliseptol Rapid | 1x daily | 1x daily | 1x daily | as required**^3^** |  |  |  |

Footnote 1: Take sample before applying product to a particular body site.

Footnote 2: When a sample turns up positive, reinitiate or continue treatment as listed for day 7 with a maximum of 3 cycles of treatment

Footnote 3: As required according to the clinical situation independent of the MRSA status.

Supplementary Table: Decolonisation protocol, contd.

| **B. Patient’s utensils** |  | | | |
| --- | --- | --- | --- | --- |
| **Utensils** | **Action** | **Days 1 to 5** | **Days 6 to 11** **^4^** | **Days 12 to 22** |
| Tootbrush and prothesis | Desinfect with ProntOral | 3x daily | 2x daily | When patient’s samples are all negative, no further actions required. When positive, repeat regime as for Days 6-11. |
| Glasses, hearing aids, hair utensils (combs etc). | Desinfect with Prontoderm | 1x daily | 1x daily |  |
| Shaving utensils | Desinfect with Meliseptol tissues | 1x daily | 1x daily |  |
|  |  |  |  |  |
| Washing dishes, bedpans, urine flasks etc. | Desinfect with Hexaquart | 1x daily | 1x daily |  |
| Other aids (walking aids etc) | Desinfect with Hexaquart | 1x daily | 1x daily |  |
| Clothing **^5^** | Wash at maximum temperature | 1x daily | 1x daily |  |
| Headcovers e.g. scarves | Wash or change | 1x on days 1 and 5 | 1x on day 10 |  |
| Footware in case of local wounds | Desinfect with Meliseptol Rapid | 1x daily | 1x daily |  |
| Towels and bed linen | Change | 1x daily | 1x daily |  |

Footnote 4: terminate actions when a complete negative series is obtained

Footnote 5: any jewelry should be desinfected on day 1 and not worn until a negative status is obtained

| **C. Patient’s environment** |  | | |
| --- | --- | --- | --- |
| **Environment** | **Action** | **Days 1 to 11** **^6^** | **Days 12 to 22** |
| Bathroom/toilet including floors | Clean/desinfect with Hexaquart | 1x daily | When patient’s samples are all negative, no further actions required. When positive, repeat regime as for Days 1-11. |
| Handles, telephone, remote control etc. | Desinfect with Meliseptol tissues | 1x daily |  |
| Bed, bedside table, surfaces in contact with skin/hair | Clean/desinfect with Hexaquart | 1x daily |  |
| Materials in contact with skin/hair (pillows etc.) | Cover with washable linen | change 1x daily |  |
| Furniture not contact with skin/hair, carpets | Vacuum cleaning | 1x per 3 days |  |
| Washable floors | Wet cleaning | 1x per 3 days |  |

Footnote 6: terminate actions when a complete negative series is obtained
